# Designing acceptable, feasible and engaging social prescribing pathways: the case of connecting from general practice to ‘Golf for Health’

**DOI:** 10.1186/s12889-026-28781-4

**Published:** 2026-07-28

**Authors:** Sharon A. Carstairs, Lynsey R. Brown, Mara van Beusekom, Gozde Ozakinci, Frank Sullivan, Andrew J. Williams, Kathryn B Cunningham

**Affiliations:** 1 School of Health Sciences, University of Dundee, Dundee, UK; 2 Population and Behavioural Sciences, School of Medicine, University of St Andrews, St Andrews, UK; 3 Division of Psychology, Faculty of Natural Sciences, University of Stirling, Stirling, UK; 4 Scottish Collaboration for Public Health Research and Policy, School of Health in Social Science, University of Edinburgh, Edinburgh, UK

**Keywords:** Social Prescribing, Physical Activity, General Practice, Community Connection, Connection Pathway, Referral Pathway

## Abstract

**Background:**

Globally social prescribing (SP) is receiving increasing attention for its role in health and social care. There is little evidence and a call for consideration on a seldom considered and hugely important aspect of SP schemes – the connection process (identification, enhancing likelihood of behaviour change, and connection). This was a novel study aiming to design an acceptable, feasible and engaging process for connecting individuals from general practice to a free golf programme in Scotland.

**Methods:**

A two-phase participatory approach drawing on the double diamond process of design was utilised in the study. Phase 1 ‘Discover to Define’ comprised an exploratory survey to determine views on potential barriers, facilitators and connection process options. Phase 2 ‘Define to Develop’ comprised online interactive workshops defining the options. Participants were key stakeholders including GP practice staff (survey *n* = 9, workshop *n* = 2), golf club staff (*n* = 10; *n* = 4), community link workers (CLW) (*n* = 2; *n* = 0), and members of the public (*n* = 9; *n* = 10). The data was synthesised using a visual mapping exercise guided by a framework of the three elements of connection. Summaries generated were validated by participants.

**Results:**

A need and benefit for flexibility in the connection process is key and a single ‘one size fits all’ pathway was not suitable for the SP of golf scheme. A connection process map outlining an approach with multiple options across an individual’s connection journey – with different combinations of options constituting different connection pathways - was designed to overcome barriers identified (e.g. uncertainty, competency, time, lack of CLW) in connecting patients to the golf programme. Suggested options not currently used in identifying, supporting and connecting the individual included practice issued lifestyle survey, text appointment and both supported and self-connection options.

**Conclusions:**

The next phase of the research programme will utilise the developed conceptual connection process map assessing the acceptability and feasibility of implementing, a flexible process of connection for the SP of golf scheme. Regarding the wider context, we encourage researchers, policymakers and practitioners working in SP across the globe to test and further develop the conceptual connection process map in designing connection pathways for their specific context(s).

**Supplementary Information:**

The online version contains supplementary material available at https://doi.org/10.1186/s12889-026-28781-4.

## Background

Evidence highlights the role of physical activity (PA) in the prevention and treatment of many long-term conditions [1] and in improving psychological well-being [2, 3]. Global public health strategic plans have continued to advocate the role of PA promotion as a ‘*best buy’* in health behaviour interventions [4, 5]. The incorporation of exercise referral/prescribing exercise into the role of healthcare professionals has played a part in PA promotion since the 1990s [6]. In recent years, golf has been considered in the development of an alternative exercise referral option [7], providing an outdoor option for PA that moves away from traditional gym-based exercise classes and which may provide greater enjoyment and intent to continue activity than indoor options [8]. Furthermore, the health benefits of golf are an area of growing research interest with evidence of associations with improved physical and mental health and wellbeing [9–11].

Healthcare settings, such as general practice, are considered key to providing opportunistic contact with a wide range and number of patients who could benefit from increasing their PA level and who could be identified and supported to become more active [12, 13]. Social prescribing (SP) is *“a holistic*,* person-centred and community-based approach to health and well-being that bridges the gap between clinical and non-clinical support and services”* [14]. This approach in healthcare covers a broad range of activities and support, such as educational sessions, dietary advice, stress management and PA opportunities, provided by the voluntary, community and social enterprises sector. SP is globally recognised to be *“delivering a social revolution in health and wellbeing”* [15] and is receiving increasing attention and acknowledgement of its role in health and social care [16–18]. However, despite some evidence on the effectiveness of SP for physical and mental health and wellbeing outcomes [19–22], varying measures of outcomes and inconsistent reporting must be acknowledged across SP [23, 24]. Furthermore, relatively little is known about which processes and methods are acceptable and effective to connect patients with community-based opportunities for health and wellbeing improvement [25, 26]. Understanding which processes and methods are acceptable and effective is of critical importance as *“the most effective opportunity in the world will not achieve its intended outcome(s) if individuals are not successfully connected with it”* [27]. The current study focused on this seldom considered and hugely important aspect of SP schemes – the connection process.

Work from a realist scoping review by the authors [28] revealed that both direct and indirect routes of connecting patients to opportunities can be employed in SP schemes. Schemes have utilised both direct and indirect routes with healthcare professionals (HCPs) directly connecting patients to PA opportunities or indirectly connecting to an intermediary (a link worker) utilising a variety of active signposting (communicating and sharing information), referral/prescription, or a combination, to connect patients with a community-based opportunity. The realist scoping review also identified three elements to the process of connection: (1) the approach to identifying eligible and willing patients who would benefit from increasing their PA levels, (2) the behaviour change strategy aiming to enhance likelihood of patient increasing their PA levels, and (3) the method of connecting patients with community-based PA opportunity [28]. Effective connection therefore requires all three elements of the process of connection to be acceptable, feasible and engaging for key stakeholders. This work provides a foundation for the current study. The aim of the study was to design an acceptable, feasible and engaging process of connection for a SP of golf scheme. The objective was to utilise a participatory approach with key stakeholders to design an acceptable, feasible and engaging process of connection from general practice to a golf programme provided by local golf clubs. The golf programme is an 8-week free programme provided by local golf clubs in Scotland in which small group coaching sessions are offered to help encourage a more active and social lifestyle (Supplementary Material File 1).

## Methods

This study was the first phase of a collaborative research programme with local golf clubs aiming to develop and test the feasibility of a SP of golf scheme. This article focuses on the initial phase of designing the connection process. A follow-on article is in development which presents findings of the next phase of study which aimed to assess the acceptability of, and feasibility of implementing, the designed process of connection and the SP of golf scheme as a whole.

### Study design

In order to design a connection process that is acceptable, feasible, and engaging for all key stakeholders, active participation of those stakeholders is essential [29]. Stakeholders in this case are: – those who could benefit from being connected, those who could identify individuals and/or connect them with opportunities, and those who could provide opportunities for participation in the golf programme. A two-step participatory approach

 drawing on the principles and process of the double diamond of design [30] was utilised. The double diamond of design is a process model that distinguishes four phases: discover, design, develop and deliver. The model helps to first focus on determining the right solution to a challenge, before further developing this solution in a fitting way. Key to this approach, and to this study, is the opportunity for the different stakeholders to be able to work and decide together, creating value for both the ‘end user’ and professionals involved [31] in delivering the SP of golf scheme. In the context of healthcare services, this collaborative way of working has been shown to transform the design and delivery of services that improve health outcomes [32, 33].

**Fig. 1:**
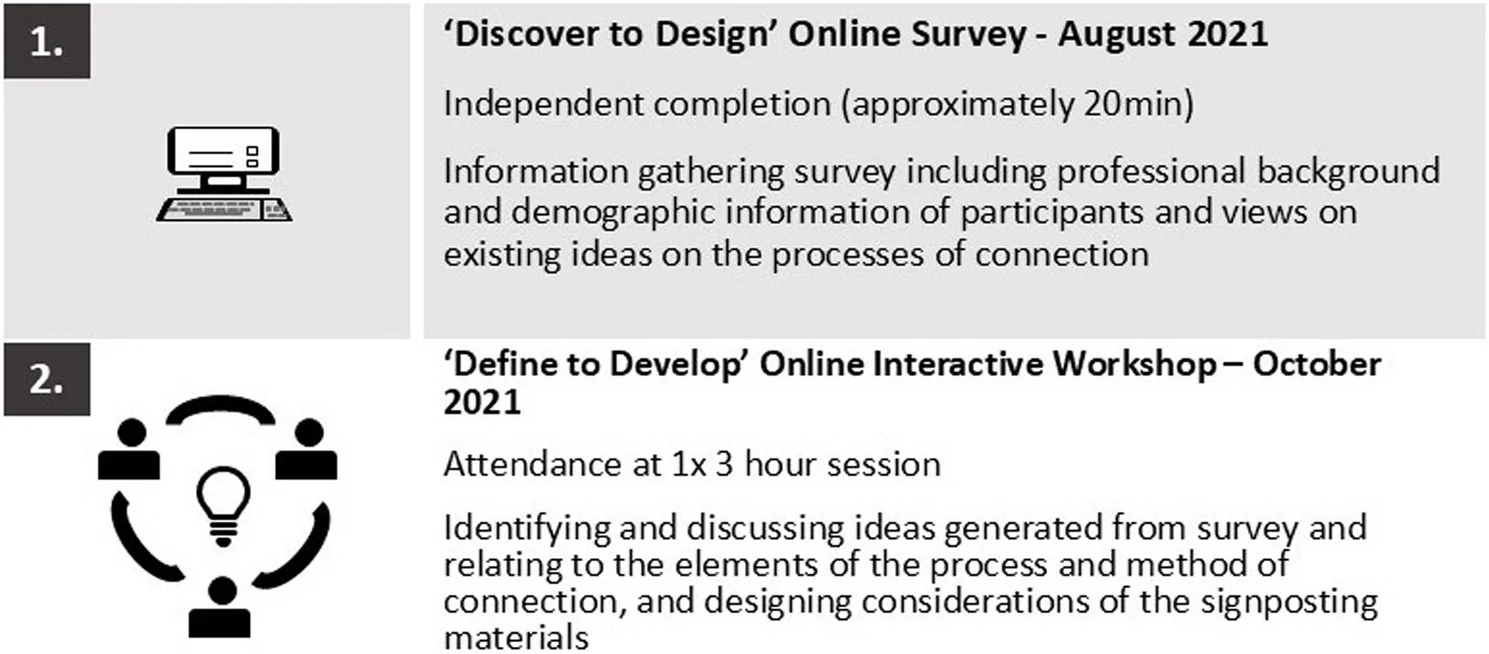
The two steps of the design approach

In the current study, a two-step approach (Fig. 1) was conducted including an initial ‘Discover to Define’ step which comprised an information gathering online survey to explore participant views on potential barriers and facilitators of the previously identified three elements of the processes of connection – identification, enhancing likelihood of behaviour change, and connection [28]. This initial step was followed by a ‘Define to Develop’ step comprising of online interactive workshops utilising collaborative and creative co-design informed strategies and tools. This two-step approach is one of many approaches which has been used in service design [34] whereby findings and questions from the initial step determined the foci for the follow-on workshops. In this instance, to refine the processes of the connection pathways and consider key barriers and facilitators of a SP of golf scheme.

### Setting

The research study was set in Fife in the East of Scotland, a county with a population of approximately 375,000 that includes urban, small town, and accessible rural areas [35]. Fife is the home of the modern game of golf and contains 50 golf courses, including the world-famous Old Course in St Andrews, within its locality [36].

### Participants & recruitment

Discussions with our wider project study collaborators identified four key groups of stakeholders for this study: General Practitioner (GP) practice staff and healthcare professionals (HCPs) working in GP practices in Fife, community link workers (CLWs) (also known as link workers and community connectors) attached to local GP practices, members of the public aged ≥ 18 years living in the local area, and golf club staff and professionals (who offer coaching) working in golf clubs across Fife. For the online survey, a target of approximately 40–50 participants with representation from each stakeholder group, was considered suitable to meet the expectation that at least 60% of participants would consent to be contacted on completion of the survey for ongoing participation in an interactive workshop [37]. Three workshops with 8–10 participants (with representation from each stakeholder group) in each was deemed sufficient to ensure manageability of participation and engagement in the sessions.

Staff members within National Health Service (NHS) general practices in the East of Scotland were invited to take part in the online survey through email invitation disseminated by the NHS Research Scotland Primary Care Network to all staff in NHS Fife practices. Members of the public were recruited via advertisement on social media (Facebook and Twitter) and through existing networks of community groups of the research team (Fife Community Advisory Council). CLWs were contacted by co-ordinator leads in Fife Health and Social Care Partnership and Fife Forum via email invitation. Email invites were sent to members of staff and professionals of Fife golf clubs via our project collaborators in Fife Golf Trust, Scottish Golf and the Professional Golfers’ Association in Scotland. Further recruitment for the workshops was required and included the use of advertisement on physical noticeboards in the community, in person promotion at GP practices, online advertising (e.g., via community groups, social media, Fife Community Advisory Council), and through existing connections of the research and collaborative team (e.g., golf club contacts). Participants were offered a high street shopping voucher for completion of the online survey (£5) and for participation in the interactive workshop (£30).

### Data collection and analysis

Data collection took place between August and October 2021. The utilisation of an online approach for this study aimed to encourage engagement and increase the likelihood of participation given the difficulty in achieving a location and time suitable for all participant stakeholders and the lasting effects of reduced face-to-face participation following COVID-19 restrictions.

#### Step 1: online survey

Each participant completed one survey which was conducted utilising the survey platform Qualtrics [38]. Separate online surveys were developed for each of the participant groups: GP practice staff and HCPs, members of public, golf club staff and professionals, and CLWs. The surveys consisted of demographic (age, gender) and background questions relevant for each stakeholder group (public: experience with golf, all other participants: role, experience with exercise referral/social prescribing) (Supplementary Material File 2). Open-ended questions were developed and utilised to explore participants’ views and opinions on the three elements of the connection process described earlier [28]. Analysis of the surveys included descriptive counts of closed-answer questions (conducted by SAC) and synthesis and mapping of the open-ended data to the three elements of the connection process framework [28] to identify what is known and what needs further exploration, and thus the foci of the interactive workshops (conducted by LRB and SAC).

#### Step 2: online interactive workshop

**Fig. 2:**
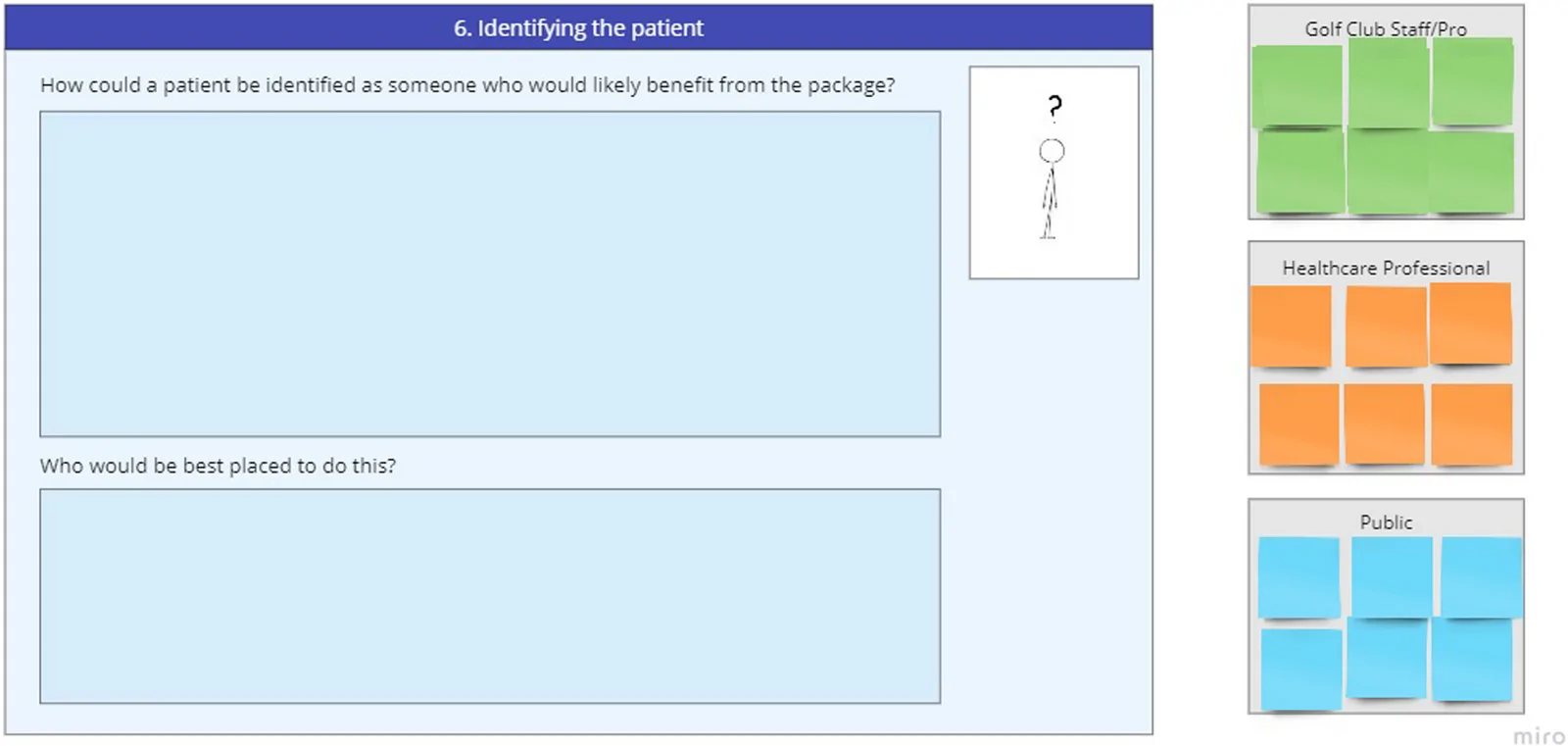
Example of interactive whiteboards for workshop

Each participant took part in one interactive participatory design workshop which was conducted utilising Zoom as a means for audio communication alongside the use of an interactive visual collaborative whiteboard, MIRO© [39]. The interactive whiteboards were designed to include a welcome and introductory task to familiarise the participants with navigating and contributing to the board by utilising pre-created sticky notes colour coded for different participant stakeholder groups (orange = GP practice staff and HCPs, blue = members of the public, green = golf club staff and professionals, yellow = CLW). Content of the online interactive workshop was derived from the findings from the online survey and included working boards on each element of the connection process (an example board can be found in Fig. 2). Suggestions for the naming of the SP of golf scheme and considerations for support to attend were included in the workshop in addition to a discussion of the design of signposting/promotional materials for the SP of golf scheme.

Participants who had registered their interest in participation but were unable to attend the organised workshops undertook a one-to-one Zoom call with a member of the research team (SAC). These participants were provided access to the interactive whiteboards from the workshops to familiarise themselves with and add their own contributions to whilst given the opportunity to discuss the content on the whiteboards.

The interactive workshops were conducted by two members of the research team (SAC, LRB) to facilitate and take observation notes during the sessions. Data collected on the whiteboards from the two workshops and one-to-one sessions and observational notes were collated and synthesised. Using a priori framework of the three elements of the connection process (identification, enhancing likelihood of behaviour change, and connection) [28] a visual mapping exercise was conducted and emergent themes of the barriers and facilitators of the connection process discussed during the workshop were synthesised from the observational notes by a member of the research team (LRB) and consensus was achieved with a second researcher (SAC). Any disagreements were resolved through discussion, with the option to consult a third team member if necessary. The visual map and a summary of the findings from the participatory workshops and one-to-one sessions was generated and validated by participants for accuracy and consensus via an online feedback survey.

## Results

### Participant characteristics

Participants in the online survey were: 13 GP practice staff and HCPs with varying professional roles (1 practice manager, 5 practice receptionists, 5 GPs, 2 practice nurses); 9 members of the public; 2 CLWs; and 10 golf club staff and professionals (5 golf professionals, 4 golf club managers, 1 golf club owner). Four participants did not complete the survey (1 practice receptionist, 1 GP, 2 practice nurses) and responses were not included in the analysis. Table 1 describes survey participant demographics. Participants in the workshops included: 2 GPs, 1 golf professional, and 10 members of the public, a further 3 golf professionals took part in the one-to-one sessions.

**Table 1:** Online survey demographics of participant groups

	Participant Group			
	Members of public (*n* = 9)	Community link worker (*n* = 2)	GP practice staff and healthcare professionals (*n* = 9)	Golf club staff and professionals (*n* = 10)
Gender				
Female	*n* = 6 (66.7%)	*n* = 1 (50%)	*n* = 7 (77.8%)	*n* = 1 (10.0%)
Male	*n* = 3 (33.3%)	*n* = 1 (50%)	*n* = 2 (22.2%)	*n* = 9 (90.0%)
Age Category (years)				
18–24	*n* = 4 (44.4%)	*n* = 0 (0.0%)	*n* = 0 (0.0%)	*n* = 1(10.0%)
25–34	*n* = 1 (11.1%)	*n* = 0 (0.0%)	*n* = 2 (22.2%)	*n* = 3(30.0%)
35–44	*n* = 0 (0.0%)	*n* = 0 (0.0%)	*n* = 4 (44.4%)	*n* = 1(10.0%)
45–54	*n* = 1(11.1%)	*n* = 2 (100.0%)	*n* = 2 (22.2%)	*n* = 3(30.0%)
55–64	*n* = 2 (22.2%)	*n* = 0 (0.0%)	*n* = 1 (11.1%)	*n* = 1(10.0%)
65+	*n* = 1(11.1%)	*n* = 0 (0.0%)	*n* = 0 (0.0%)	*n* = 1(10.0%)
Physically Active for 30 min or more (Days)				
1	*n* = 0 (0.0%)			
2	*n* = 1(11.1%)			
3	*n* = 2 (22.2%)			
4	*n* = 2 (22.2%)			
5	*n* = 2 (22.2%)			
6	*n* = 0 (0.0%)			
7	*n* = 2 (22.2%)			
Physical Activity Intention				
I never think about doing more physical activity	*n* = 0 (0.0%)			
Sometimes I think about doing more physical activity	*n* = 5 (55.6%)			
I have decided to do more physical activity	*n* = 0 (0.0%)			
I am already trying to do more physical activity	*n* = 3 (33.3%)			
I have already increased my physical activity	*n* = 1(11.1%)			
Golf Experience				
Never played golf in any form	*n* = 2 (22.2%)			
Novice golfer – played some putting, driving range or tried golf	*n* = 7 (77.8%)			
Infrequent golfer – play on a full course more than once a month but less than 12 times in a year	*n* = 0 (0.0%)			
Regular golfer – play on a full course at least once a month or more than 12 times in a year	*n* = 0 (0.0%)			
Role				
			Practice manager *n* = 1 Practice receptionist *n* = 4 General practitioner *n* = 4	Golf professional *n* = 5 Golf club manager *n* = 4 Golf club owner *n* = 1

### Naming of the golf programme

**Fig. 3:**
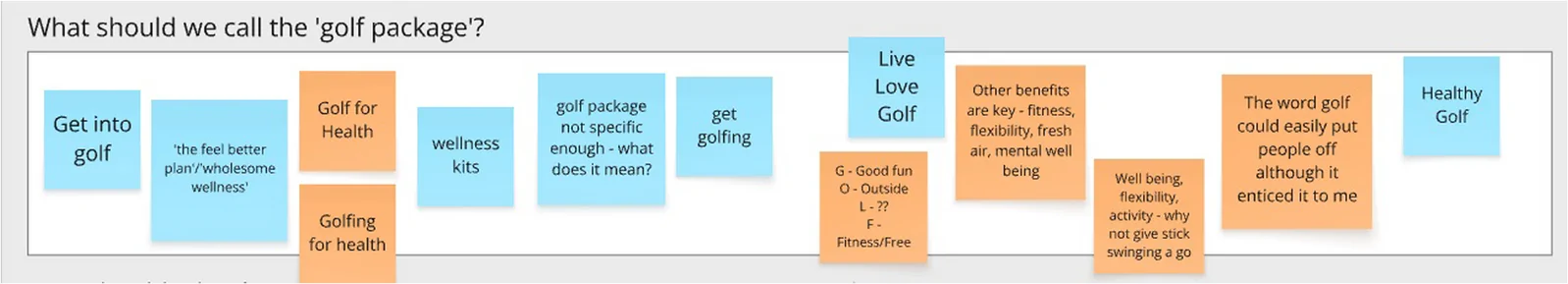
Naming suggestions from workshop one for the SP of golf scheme

During the workshops and sessions, we took the opportunity to ask participants to suggest and discuss a name for the golf programme offered by local golf clubs in this SP of golf scheme. From the suggestions provided by participants (Fig. 3 shows board from workshop one), *Golf for Health*, was suggested by the participants and following research team discussion, was adopted by the researchers and collaborators on the project.

### Developed connection pathways

**Fig. 4:**
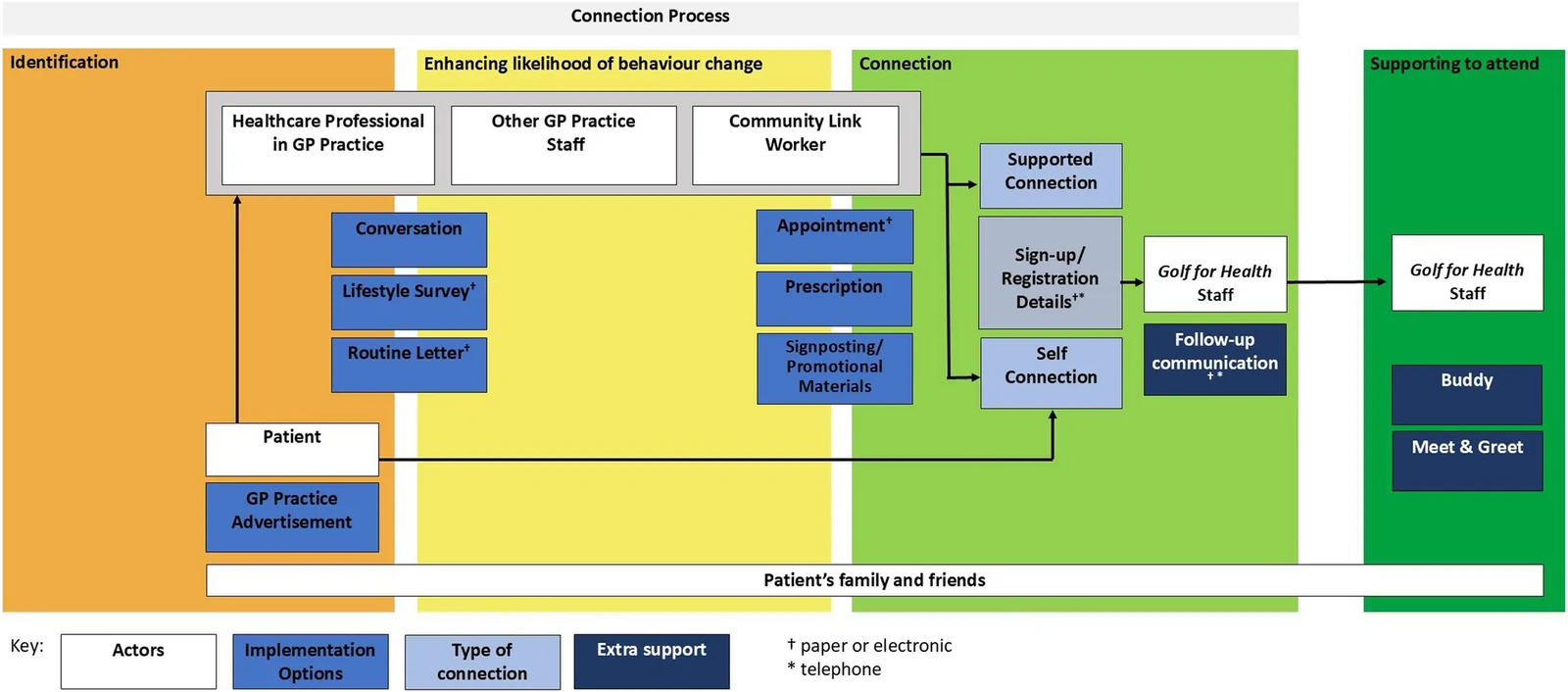
Map of the designed acceptable, feasible and engaging options within the connection process from GP practices to *Golf for Health*

During the design process it became clear that there was not a ‘one size fits all’, neither in thinking about the process as a whole nor in thinking about any of the three elements of the process (identification, enhancing likelihood of behaviour change, connection). It became evident that what was required was a map of acceptable, feasible and engaging options regarding each element of the process (Fig. 4). These options concerned (1) the actors – the people who have an active role in the connection process, (2) the implementation options – the ‘ways of doing’ each element, and (3) the type of connection – supported connection or patient self-connection. This would enable a flexible approach in which appropriate options could be selected to create one or more pathways tailored to each specific context.

### Actors in the connection process

A number of key people can have an active role in the connection process (herein named actors): HCPs and other GP practice staff, CLWs, community PA organisers (in this case golf club staff and professionals), and patients. The workshop discussed the role these actors could play in aiding the identification of eligible and willing patients who could benefit from increased PA levels, help enhance the likelihood of behaviour change, and aide patients in the successful connection to *Golf for Health*, and highlighted that the family and friends of patients may also have an important role throughout these elements of the patient journey.

### Implementation options of the connection process

A variety of implementation options were recognised to have potential in identifying eligible and willing patients, enhancing the likelihood of behaviour change, and connecting patients to *Golf for Health*. These options could be utilised individually or in combination with others and could have dual purposes across the elements of the connection process. They included: GP practice issued lifestyle surveys and routine letters, prescriptions, conversations, signposting and promotional materials, and appointments (see Table 2 for more detail). Amongst the options, conversation, and GP practice advertisement, signposting and promotional materials were identified as approaches already regularly utilised in Fife GP practices and the use of a prescription is something that some HCP participants were aware of being utilised in other health board areas. These implementation options could then be followed by online, paper or over the telephone form completion for signing-up to attend.

**Table 2:** Description of the varying implementation options

Implementation Options	Description of utilisation
*Conversation* ^†^	Identification of need and the benefit of increased PA may be ascertained during an HCP and patient consultation through meaningful conversation. This conversation approach establishes current PA levels and activities which could then be followed by a person-centred approach to provide PA advice and help support behaviour change. A conversational approach could therefore be part of the first two elements of the connection process, identifying need and enhancing likelihood of behaviour change, but may also be combined with the use of other implementation options such as promotional materials to help signpost and support patients towards *Golf for Health*.
*Lifestyle survey*	A GP practice issued lifestyle survey could provide notifications to HCPs regarding those patients who could benefit from improving their PA. Alternatively, completion of the lifestyle survey could highlight to the patient their current PA level and the potential benefit of increasing PA.
*Prescription*	A written instruction provided by the HCP or CLW for a patient to be authorised to attend a treatment – in this case the treatment is participation in *Golf for Health*.
*Routine letter*	A GP practice/HCP issued letter, linked to a previously identified health condition and monitoring of condition, could be utilised to encourage reflection and assessment of PA level and include element(s) to support behaviour change which could be issued alongside promotional materials to signpost to *Golf for Health* or could trigger a follow-on conversation with HCP/CLW to support behaviour change.
*GP practice advertisement and* *signposting and promotional materials* ^†^	A variety of materials to encourage self-identification of need/benefit to PA improvement, which could additionally enhance likelihood of behaviour change individually or in combination with the identification methods and other implementation options. Materials could include posters, leaflets, booklets, video clips, located on GP practice noticeboards and TV/digital noticeboard, practice linked social media and website pages.
*Appointment (to attend)*	A letter or text message could be issued to provide an appointment to attend *Golf for Health*. The issuing of an appointment can enhance the likelihood of behaviour change as a motivational prompt, but the appointment itself is a method of connecting to the opportunity as it is arranging attendance.
*Registration/sign-up form*	Patient information for contact and registration could be provided in a form structure that can be completed online, via hardcopy paper format, or conducted over the telephone in a call to *Golf for Health*.

† Denotes implementation options that were identified as currently or having been utilised in GP practices in Fife

**Fig. 5:**
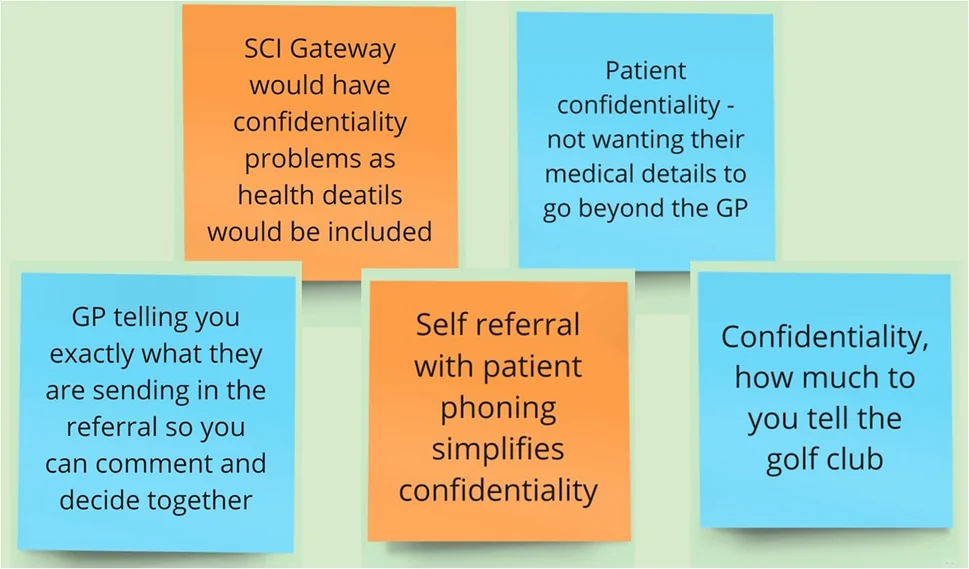
Selection of points raised on data confidentiality. Note: SCI Gateway mentioned in sticky note refers to an NHS Scotland technology system for health and social care professionals to access and share confidential information

Discussion across this study highlighted that strong consideration is needed on how patient data is shared in the process of connection and includes registration. Limitations and difficulties in linking all third sector community groups and opportunities, such as *Golf for Health*, to the healthcare system were raised alongside the issue of data confidentiality. To overcome and minimise concern over data confidentiality, limiting the amount of patient information passed on and providing options for patients in how they are signed-up for *Golf for Health* were key (see Fig. 5).

### Designing signposting and promotional materials for the SP of golf scheme ‘Golf for Health’

During the workshops, participants were asked to consider various aspects in designing signposting and promotional materials that could be utilised in the connection of patients to the SP of golf scheme *Golf for Health*.

**Fig. 6:**
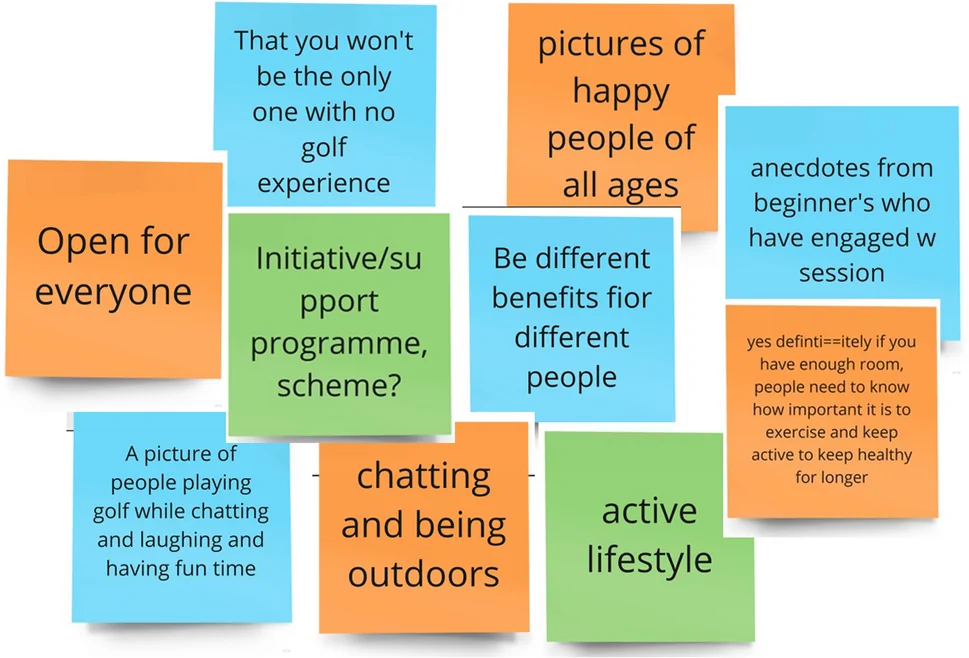
Suggestions from workshops on aspects to consider in signposting and promotional materials

Reflection on what is needed in the promotional materials included ensuring that messaging is clear and concise, in particular indicating that the scheme was ‘free’, showing the opportunity is inclusive and open to all, and highlighting the supportive nature of the SP of golf scheme *Golf for Health* and the social, physical and mental health benefits to be gained from participation (See Fig. 6).

### Types of connection

**Fig. 7:**
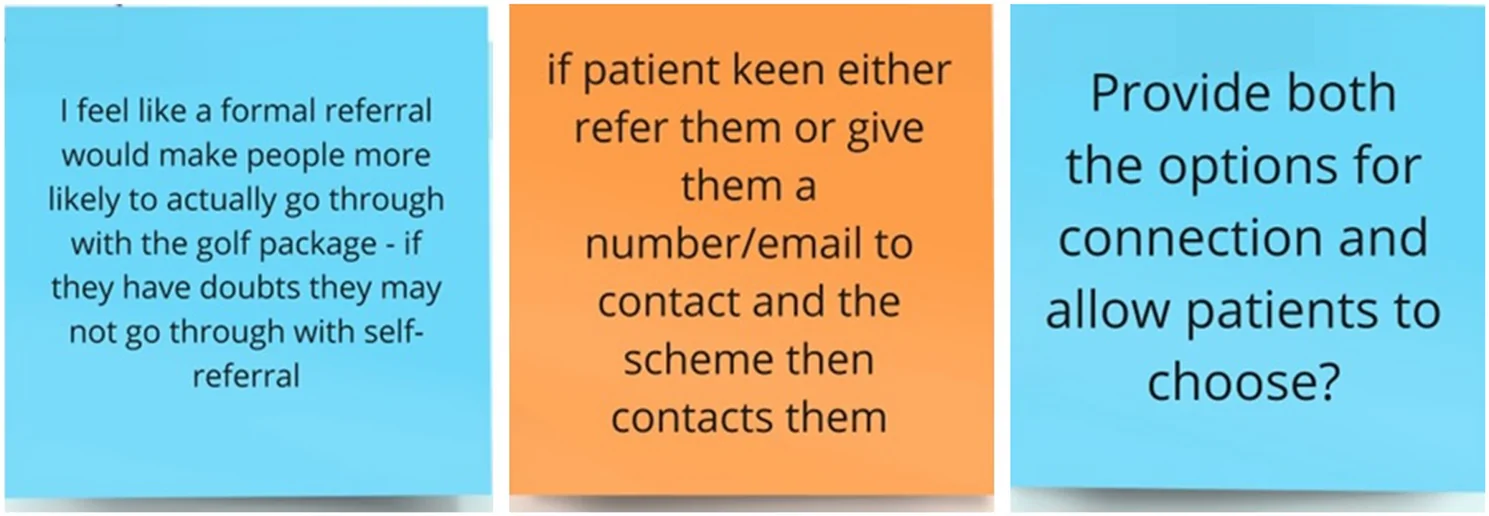
Selection of points raised in relation to flexibility needs of pathway

It was apparent during this study that providing a flexible approach that includes implementation options (as described above) and connection options (supported connection or self-connection) can potentially help to alleviate the barrier of patient motivation. Providing options for supported connection (where HCPs, other GP practice staff, CLWs help patients complete sign-up) and self-connection (where patient completes/conducts sign-up on their own) can help meet differing individual needs and levels of competency (see Fig. 7).

### Additional support for patients

**Fig. 8:**
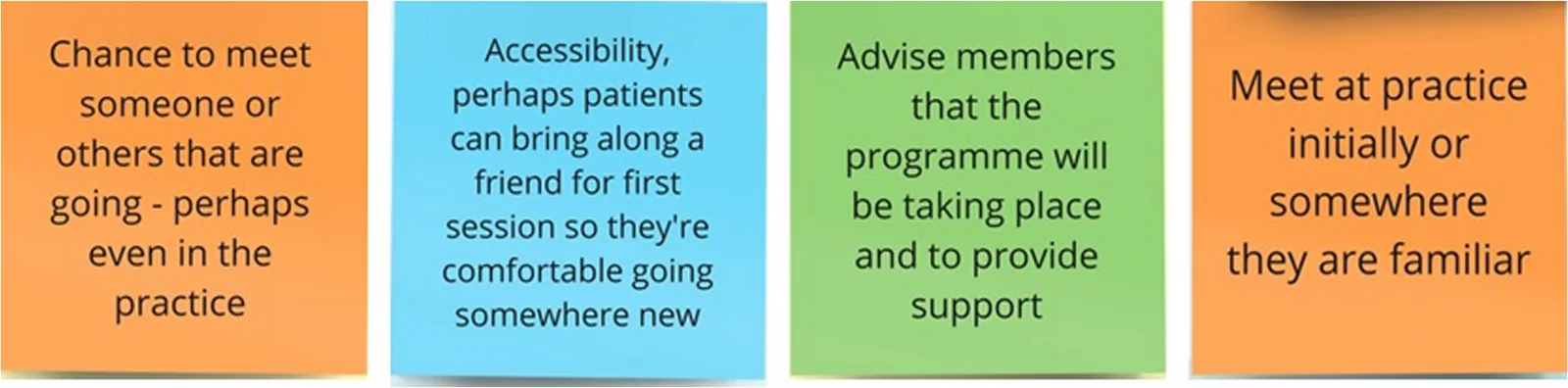
Selection of points raised on additional support options for patients to attend

During this study, participants suggested providing opportunity in the pathways to further support patients to engage with the SP of golf scheme. This additional support includes follow-up communication issued by the *Golf for Health* organisers once successful connection has occurred. This can potentially help keep patients motivated and feel included and a part of the scheme even before attending their first session. This could also be achieved by inviting patients to a meet and greet session alongside fellow patients on the programme and adopting and promoting a buddy scheme in which patients could bring along family member or a friend or be linked to a volunteer at the golf club (Fig. 8). These additional support options could provide opportunities for familiarity and social support to alleviate anxiety.

## Discussion

The findings highlight that flexibility in the connection process is key and that a single ‘one size fits all’ pathway was not suitable for the SP of golf scheme. In response to the identification of a need for a flexible approach to connection, a conceptual connection process map outlining such an approach was designed for a SP of golf scheme in this participatory approach study. The connection process map delineated multiple acceptable, feasible and engaging options across each element of the connection process (identification, enhancing likelihood of behaviour change, and connection). As such, pathways could be tailored to overcome potential barriers and meet the differing needs, levels of competency and motivation of those involved. Potential actors in the connection process from GP practice to *Golf for Health* not only included the key stakeholders for this SP of golf scheme: patients, GP practice staff and HCPs, CLWs, and the golf club staff and professionals, but also the patients’ family and friends. A variety of options for implementation were identified, for use on their own or in combination with others, to progress a patient’s connection in the SP of golf scheme. Some were already in use in Fife: signposting/promotional materials, conversation, with an awareness of the use of prescription in other health boards, but novel ideas also emerged: GP practice issued lifestyle survey and routine letter, appointment (text), leading to registration/sign-up form completion. Making the connection to *Golf for Health* could be achieved via supported connection by HCP/GP practice staff/CLWs or by the patient themselves (self-connection), providing control and autonomy to take the step towards successful connection. Providing the option for a supported connection process and the opportunity for additional support in attending and participating in *Golf for Health* in the form of follow-up communication, meet and greet and buddy schemes were also identified as useful options to facilitate connection.

The need for a flexible pathway found in this study reflected the complexity of meeting the needs of all stakeholders. At the patient level, providing options for self or supported connection in some or across all elements of the connection process can help facilitate connection across different motivational, competency levels and needs of patients. Secondly, variety in the implementation options that can be utilised in the connection of patients to *Golf for Health* demonstrates the need to provide the opportunity for individual GP practices, *Golf for Health* providers and patients to implement a SP scheme that is acceptable, engaging and feasible, and which accommodates the resources available to them (including presence of CLWs). These findings not only provide insight for the local context for this SP scheme but adds to the ongoing debate and consensus of the definition [14] and the landscape of implemented SP schemes [40, 41], particularly with regards to signposting and self-connection (self-referral) as an approach [14, 42]. Furthermore, the flexibility in connection pathways to involve CLWs or not [28, 40] is another aspect for consideration and debate. During the course of this study, the authors discovered that small numbers of CLWs were employed in the Fife area with many working only in selected GP practice areas and with specific roles for supporting patients with particular health conditions. The low number of CLWs working across selected GP practices in Fife is representative of the roll-out of the 2016 Scottish Government plans for SP link workers [43] where CLW roles and implementation of SP varies greatly across different healthcare services [44, 45]. The provision of pathway options that employ HCPs and GP practice staff as well as or in the absence of CLWs to help patient connection provides a shared role responsibility and wider opportunity for supporting patients, in this case in improving their PA levels. This shared role in SP can be a key aspect not only when there are limitations in the availability of CLWs in place in the locality but also limitations in HCP time [46, 47]. Furthermore, the present study revealed consideration of the option for a supportive role for family and friends throughout an individual’s SP journey across some or even all elements of the connection process: identification, supporting behavioural change, connecting and registering and attending. Our findings highlight the importance of recognising that SP may require flexibility in its connection approach to be able to respond to the diverse needs of all stakeholders based on individual needs, local contexts and addressing the wide variation and availability of resources.

The present study illustrated that for many GP practices and HCPs the option for a shared role with CLWs to help connect patients to SP schemes may be limited or unobtainable, thus utilising multiple implementation options and having a SP pathway with extra opportunities for support may make a difference towards successful connection in these contexts and for certain populations of the public. Social support has been considered as playing a potential role in facilitating individual behaviour change [48] and the use of buddy schemes in lifestyle interventions has become a more recent consideration [49, 50]. A pathway that incorporates social support opportunities, such as follow-up communication from the organisers, meet and greet sessions prior to beginning *Golf for Health*, and buddy schemes, was considered in the present study. The inclusion of a social support step in the connection process of a SP of PA scheme may provide the opportunity to aid and encourage patients in their journey towards successful connection and ultimately may impact on successful connection and retention in PA [24]. More widely, inclusion of social support, such as buddy schemes, has been identified as a key ingredient to SP for mental health [51] and cultural interventions [52].

In a scoping review of SP intervention components, Sandhu et al. [53] found that very few studies reported on the implementation options utilised for connection and urged the consideration of surveying SP programmes to allow comprehensive evaluation of all components of these interventions. The implementation options identified in the review including referral form, prescription pad, online referral system and information materials, provide some insight to the connection methods being utilised in SP schemes and interventions however, a lack of detail of the process of connection across the vast reporting of SP is evident [28, 53]. The present study has identified further potential implementation options in addition to those identified in Sandhu et al.’s review [53] and those being utilised currently in GP practices that may be implemented, such as GP practice issued lifestyle survey and letter or the use of text appointments. Although detailed in the present study for identifying and supporting individuals who would benefit from being more physically active, these options (lifestyle survey and letter) may also have wider application in areas such as diet, social isolation, mental health and wellbeing. Testing of this conceptual connection process map and evaluating the effectiveness of the different implementation options found in the present study is the next step for the current SP of golf scheme and will be key in understanding their role in the connection process and supporting individuals to change behaviour [25, 27, 28]. This evaluation is critical, and supports current thought that in order to evaluate SP schemes there needs to be clear and detailed instruction and reporting of ‘who, what, when, and how’ [54, 55] concerning each element of the SP connection process: identification, enhancing likelihood of behaviour change, connection [25, 27].

With SP becoming a key component in government health and social care plans [17, 18] and recognition of the impact of SP of PA schemes in providing improvement in PA levels, wellbeing and social interaction [24], it is essential that individuals who may benefit from SP schemes are effectively connected to them. This was a novel study focusing on a seldom considered and hugely important aspect of SP schemes – the connection process. Utilising an interactive participatory approach to design and map out the options for the three elements of the connection process from GP practices to a local community-based PA opportunity, this work identified the need for a flexible process of connection in the case of connecting to *Golf for Health*. However, it is reasonable to expect that the need for a flexible approach to connection with multiple options will also apply in other contexts, e.g. in other countries, healthcare systems and in other SP schemes, therefore our findings and conceptual pathway map have global significance and wider implications to the SP field. We encourage those working in the field of SP to test, tailor or further develop this conceptual connection process map in designing connection pathways for their specific context(s).

The interactive, engaging participatory design sessions with all key stakeholder groups provided this study an invaluable opportunity to build a relationship and share power in designing a journey for connecting patients to a local community-based opportunity. This was evidenced through the naming of the golf programme - *Golf for Health* - by the participants, in the views and idea generation of signposting/promotional materials, and in the designing a conceptual connection pathway that works for all. Feedback from the validation survey revealed all respondents felt the interactive design process allowed them the opportunity to contribute. Golf has historically been a male-dominated activity and despite increases in female participation in golf in recent years, there remains a gender imbalance to golf participation [56, 57]. One strength of the current study was our recruitment of both genders. The present study did not aim to study any gender-related issues or considerations in respect to the connection process; however, inclusivity as a whole was raised. The workshops provided a unique opportunity to help design materials to target audiences who might otherwise be against or unsure of golf as an opportunity to improve PA levels and mental health and wellbeing. Nevertheless, we acknowledge that there may be gender-related perceptions of the suitability of a SP of golf scheme by individuals and other actors in the identification, support and connection process. One limitation of the study was a failure to meet target sample sizes for both the survey and workshops which may impact on the representativeness of our findings. In particular, the authors acknowledge the lack of involvement by CLWs and low numbers of GP practice staff and HCPs from a variety of different roles across both steps of the participatory design approach as a limitation in this study. Engagement by health and social care staff was difficult due to the time constraints of participating in the whole participatory approach [58] and given the low numbers of CLWs working in the local area [45]. The provision of one-to-one sessions with participants who could not attend the organised workshops provided an extra opportunity to engage stakeholders within the time constraints of the study funding, nevertheless providing a more flexible approach where more engagement points and asynchronous opportunity is provided for people to participate without the restriction of time [59] could be a consideration for future work. The authors additionally acknowledge that a targeted approach to recruiting members of the public may have been of benefit for this study to engage those people who would benefit the most from increased PA. Identification of these individuals, however, was amongst one of the key questions in this study and thus we included a broader scope towards the public’s view on how connection could be achieved. Furthermore, the lack of a targeted approach to recruit members of the public specifically without any pre-existing interest in physical activity or golf could result in a potential bias in our participant sample and may influence the perceived acceptability and feasibility of the pathways designed. Finally, it should be highlighted that the connection process map designed from this participatory approach study is conceptual in nature and has been considered for primary care SP in Scotland in an area with a cultural history with golf. The acceptability and feasibility of this conceptual connection process map require further testing in a variety of real-world settings and contexts to ensure the transferability and appropriateness for practical application across regions, systems and SP activities.

## Conclusions

The aim of the study has been achieved in the designing of a flexible connection process for a SP of golf scheme from general practices to *Golf for Health* via the development of a conceptual connection process map with options for each of the three elements of the connection process. Regarding the next steps, the second phase of the research programme will assess the acceptability of, and feasibility of implementing, the flexible process of connection and the SP of golf scheme as a whole. Regarding the wider context of SP, we call on researchers, policymakers and practitioners working in the field across the globe to consider the likely need for, and benefit of, a flexible connection approach. We also encourage them to test and further develop this conceptual connection process map in designing connection pathways for their specific context(s).

## Supplementary Information

Supplementary Material 1.

Supplementary Material 2.

## Data Availability

The datasets generated and/or analysed during the current study are available from the corresponding author on reasonable request.
