# Supplementary material for: Designing acceptable, feasible and engaging social prescribing pathways: the case of connecting from general practice to ‘Golf for Health’
Source: BMC Public Health. 2026 Jul 28;26:3045. doi: 10.1186/s12889-026-28781-4 (PMC13692886; doi:10.1186/s12889-026-28781-4)
Supplement: Supplementary file 1 [file 12889_2026_28781_MOESM1_ESM.docx]

**Supplementary Material File 1**

What is the golf programme *Golf for Health*?

The *Golf for Health* pilot in Fife will utilise a programme very similar to the tried and tested Get into Golf model offered by golf clubs. Get into Golf is an existing model of introducing people to the game. It is important to recognise that newcomers to the game generally do not want to spend lots of money, get bogged down by lots of rules, or to feel intimidated. They also do not want to be overwhelmed with lots of golfing jargon.

Structure:

- Coaching programme (e.g. 8 participants) over 8 weekly sessions to meet the activity needs of the participants and the group.
- Incorporation of at least 30 minutes walking into each session (e.g. walking between shots, on course activities - to introduce the course/layout/rules or on course play/practice).
- Coaches and hosting golf clubs provide equipment for participants to use.
- When required coaches should draw the groups attention to relevant educational information supplied to help support getting more active.
- Coaches and hosting golf clubs provide volunteers/buddy’s during the programme and communicate with participants prior to the delivery of the group coaching sessions
- Participants are provided social opportunities and encouraged to use golf club and facilities between sessions.
