# Supplementary material for: Designing acceptable, feasible and engaging social prescribing pathways: the case of connecting from general practice to ‘Golf for Health’
Source: BMC Public Health. 2026 Jul 28;26:3045. doi: 10.1186/s12889-026-28781-4 (PMC13692886; doi:10.1186/s12889-026-28781-4)
Supplement: Supplementary file 2 [file 12889_2026_28781_MOESM2_ESM.docx]

**Supplementary Material File 2**

**Phase 1 ‘Discover to Define’ exploratory surveys for each stakeholder participant group**

**MEMBERS OF THE PUBLIC SURVEY**

The aim of this research project is to design connection pathways from GP practices to golf packages offered by local golf clubs in Fife for adults who would benefit from improving their physical activity levels. We are keen to hear your views on these golf packages and how GP patients can be connected with them.

The packages offered by local clubs aim to increase physical activity levels and improve physical and mental health and wellbeing for those adults new to golf and those who have not played in some time. Access to equipment, appropriate coaching sessions and golf activities facilitated by a qualified coach will be provided. A buddy scheme will be central to these packages to help participants feel supported. Social events will allow the participants to build bonds and feel part of the club. These buddy and social links will help to encourage participants to continue with their golf experience once the package has ended. Clubs may also offer participants access to follow-on sessions or activities and membership offers to maintain their engagement.

We would be grateful if you would answer the following questions. You are free to leave any question that you do not wish to answer.

Section 1: We want to make sure we get a wide range of perspectives. Therefore, we will ask you a few questions about yourself and your experiences.

1.	What is your age (years)?

18-24

25-34

35-44

45-54

55-64

65-74

75+

2.	How would you describe your gender?

Female

Male

Other gender identity: ___________________________

Prefer not to answer

3.	In the past week, how many days have you been physically active for a total of 30 minutes or more? Note: Physical activity can include walking, cycling, gardening, housework and exercise or sport. Anything that gets the heart rate or breathing rate up a little.

One

Two

Three

Four

Five

Six

Seven

a)	If four days or less, have you been physically active for at least two and a half hours (150 minutes) over the course of the past week?

Yes

No

4.	Which of the following statements best describes how you feel right now?

I never think about doing more physical activity

Sometimes I think about doing more physical activity

I have decided to do more physical activity

I am already trying to do more physical activity

I have already increased my physical activity

5.	What benefits do you think you would get from being more physically active?

[text box]________________________

6.	What experience do you have with golf?

Never played golf in any form

Novice golfer – played some putting, driving range or tried golf

Infrequent golfer – play on a full course more than once but less than 12 times in a year

Regular golfer – play on a full course at least once a month or more than 12 times in a year

7.	Have you ever had any experience with being referred to a physical activity opportunity or a community-based group from your GP practice or other healthcare professional?

Yes

No

a)	If yes, please give a brief description of how this worked i.e. GP/health professional completed form and referral team contacted you, GP/health professional gave you leaflet/letter and you made contact with referral team

[text box]________________________

b)	If yes, what worked well/didn’t work well?

[text box]________________________

Section 2: The packages offered by local clubs aim to increase physical activity levels and improve physical and mental health and wellbeing for those adults new to golf and those who have not played in some time. Access to equipment, appropriate coaching sessions and golf activities facilitated by a qualified coach will be provided. A buddy scheme will be central to these packages to help participants feel supported. Social events will allow the participants to build bonds and feel part of the club. These buddy and social links will help to encourage participants to continue with their golf experience once the package has ended. Clubs may also offer participants access to follow-on sessions or activities and membership offers to maintain their engagement.

8.	What do you think should be included in the golf package to achieve the aim of increasing physical activity levels and improving physical and mental health and wellbeing? [you can select multiple options]

group coaching session

putting activities

driving range activities

buddy scheme

social activities/events

provision of golf equipment during package

other:____________

9.	What do you think should be provided after the golf package is finished? [you can select multiple options]

follow-on golf sessions

follow-on invites to social events

membership offers

other: ___________

Section 3: There are different ways in which people could potentially be linked with the golf packages. One way is through the GP practice. A challenge is finding the people who would benefit most from this programme. We will now ask you some questions about this.

10.	Would you be happy for your GP practice to use your electronic patient health record to identify you as someone who could potentially benefit from being linked to a community-based golf package or physical activity opportunity?

Yes

No

Unsure

a)	If unsure, please provide a brief reason:__________

11.	Would you be happy for your GP or Practice Nurse to raise the issue of physical activity with you during an appointment?

Yes

No

Unsure

a)	If unsure, please provide a brief reason:__________

b)	How, if at all, would this differ depending on the reason you were attending?

[text box]________________________

12.	In some cases, link workers can help GP practices connect patients with other types of services and groups in the community. For example, a patient who a GP feels could benefit from taking part in some physical activity to improve their health would be referred to the link worker to discuss some physical activity options available in their area. The GP practice and/or link worker can identify how active someone is and help advise on what physical activity support is needed by using the following question tool [link to questions].

Would you be happy for GP/GP practice staff or a link worker asking you these questions to see if you would benefit from increasing your physical activity and taking part in a community-based golf package?

Yes

No

Unsure

a)	If unsure, please provide a brief reason:__________

13.	Would you be happy to fill out these questions yourself to assess your PA level and suitability for a referral to a community-based package?

Yes

No

Unsure

a)	If unsure, please provide a brief reason:__________

Section 4: There are different ways in which people could be encouraged and helped to increase their physical activity levels by taking part in a community-based golf package. We keen to hear your views about this.

14.	How do you think a GP/health professional could encourage patients to go along to a community-based golf package?

[text box]________________________

Section 5: There are several potential ways in which GP patients could be linked with a golf package. We would appreciate your views about this.

15.	How do you think GP patients would like to find out about a community-based golf package? [you can select multiple options]

Through an advert in the practice/on the practice website

Through a personalised postal letter from their GP practice

Through a conversation with their GP/health professional during a consultation

Through a conversation with a link worker following referral/signposting to the link worker by their GP/health professional

Other:________

16.	If you were identified as someone who was eligible for and would benefit from a community-based golf package, would you be happy for GP/health professional/GP practice/link worker passing on your details to the people running the golf package for them to contact you?

Yes

No

Unsure

a)	If unsure, please provide a brief reason:[text box]________________

17.	What would you prefer?

a)	GP/health professional/link worker passes on your details to the people running the golf package for them to contact you

b)	GP/health professional/link worker gives you contact details of the people running the golf package and you contact them yourself

Please provide a brief reason for your choice: [text box]________________________

18.	Do you have any further ideas on how GP patients could be connected to community-based golf packages?

[text box]________________________

END OF SURVEY NOTE REGARDING STEP 2: CO-DESIGN SESSION

We thank you for your participation in this research project.

Please provide an email address for us to send you a £5 digital thank you voucher for your participation. This will not be used for any other purpose.

________________________________________________________________________________

If you would like to find out more information about taking part in the next step of this research and are happy for us to contact you using the email address you have provided then please tick this box consenting for us to contact you with further information	⃣

**GP STAFF SURVEY**

The aim of this research project is to design connection pathways from GP practices to golf packages offered by local golf clubs in Fife for adults who would benefit from improving their physical activity levels. **We are keen to hear your views on these golf packages and how GP patients can be connected with them.**

The packages offered by local clubs aim to increase physical activity levels and improve physical and mental health and wellbeing for those adults new to golf and those who have not played in some time. Access to equipment, appropriate coaching sessions and golf activities facilitated by a qualified coach will be provided. A buddy scheme will be central to these packages to help participants feel supported. Social events will allow the participants to build bonds and feel part of the club. These buddy and social links will help to encourage participants to continue with their golf experience once the package has ended. Clubs may also offer participants access to follow-on sessions or activities and membership offers to maintain their engagement.

We would be grateful if you would answer the following questions. You are free to leave any question that you don’t wish to answer.

**Section 1: We want to make sure we get a wide range of perspectives. Therefore, we will ask you a few questions about yourself and your experiences.**

1. What is your professional role?

General practitioner

Practice nurse

Practice manager

Practice receptionist

Other: ________

2. What is your age (years)?

18-24

25-34

35-44

45-54

55-64

65-74

75+

3. How would you describe your gender?

Female

Male

Other gender identity: ___________________________

Prefer not to answer

4. What is the usual care that you or your practice employs for physical activity promotion in patients?

Poster Adverts in practice waiting area

Meet and greet/in person adverting of local PA options at practice

Staff verbally ask about physical activity levels and discuss health benefits during consultation

Staff verbally promote local/available physical activity opportunities during consultation

Staff promote physical activity by providing leaflets/self-referral materials

Staff promote physical activity by discussing and completing referral forms

Other:_______

5. How, if at all, do you assess a patient’s activity level?

[text box] ___________________________

6. Have you/your practice had any experience with referring patients to an exercise referral/social prescribing scheme?

Yes

No

If yes, please briefly describe how patient was linked to the exercise referral/social prescribing scheme (i.e. discussion during consultation then provided leaflet with details for opportunity, patient approaches reception staff after seeing poster/advert in waiting area)

[text box] ___________________________

**Section 2: The packages offered by local clubs aim to improve physical health and wellbeing for those adults new to golf and those who have not played in some time. Access to equipment, appropriate coaching sessions and golf activities facilitated by a qualified coach will be provided. A buddy scheme will be central to these packages to help participants feel supported. Social events will allow the participants to build bonds and feel part of the club. These buddy and social links will help to encourage participants to continue with their golf experience once the package has ended. Clubs may also offer participants access to follow-on sessions or activities and membership offers to maintain their engagement.**

7. What do you think should be included in the golf package to achieve the aim of increasing physical activity levels and improving physical and mental health and wellbeing? [you can select multiple options]

group coaching session

putting activities

driving range activities

buddy scheme

social activities/events

provision of golf equipment during package

other:____________

8. What do you think should be provided after the golf package is finished? [you can select multiple options]

follow-on golf sessions

follow-on invites to social events

membership offers

other: ___________

**Section 3:** **There are different ways in which people could potentially be linked with the golf packages. One way is through the GP practice. One challenge is identifying the people who would benefit most from the golf packages. We will now ask you some questions about how you think this could be done.**

9. Could screening of patient health records be used to identify potential patients for connection to community-based golf packages?

Yes

No

Unsure

i. What might make this difficult in practice?

[text box] ___________________________

ii. What could make this easier?

[text box] ___________________________

iii. Who would conduct this screening?

[text box] ___________________________

10. Could patients’ eligibility for connection to community-based golf packages be checked when a patient attends a consultation?

Yes

No

Unsure

i. What might make this difficult in practice?

[text box] __________________________

ii. What could make it easier/what would help you with this?

[text box] ___________________________

iii. Who should do this?

[text box] ___________________________

11. The Scottish Physical Activity Screening Questionnaire (Scot-PASQ) is a standardised tool that provides a framework for meaningful physical activity conversations between health or social care professionals and people in their care. It helps identify how active someone is and informs what physical activity support is needed [add link to doc].

Are you familiar with this tool?

I have never seen it before

I have seen it but not used it myself in my professional role

I have used it in my professional role to identify people’s physical activity levels

Other: [text box]_____________________

12. How best could this tool be integrated in your normal workflow? (e.g. online/paper, practice team administered, or patient self-administered, timing, etc.)

[text box]_____________________

13. Do you have other thoughts on how to identify eligible patients for a community-based golf package?

[text box]_____________________

**Section 4:** **There are different ways in which people could be encouraged and helped to increase their physical activity levels by taking part in a community-based golf package. We keen to hear your views about this.**

14. What, in your experience, are effective communication strategies for promoting physical activity with patients?

[text box]_____________________

15. Do you have any suggestions and ideas about what techniques could be helpful for trying to motivate a patient to change their physical activity behaviour and then getting them to turn motivation into action?

[text box]________________________

16. What, if anything, would stop you from discussing a community-based golf package for physical activity with patients? [select all that apply]

Belief that it is not my role

Belief that a golf package will not increase physical activity levels and improve physical and mental health and wellbeing

Lack of time to discuss in consultation

Uncertainty about how to raise in consultation about health issue unrelated to physical activity

Belief that it is unsuitable for discussion in consultation about health issue unrelated to physical activity

Belief that patient would not be able to take part in a golf package due to health issues

Belief that patient would not be able to take part in a golf package for other reasons

Belief that patient would not be interested in taking part in a golf package

Lack of knowledge about suitability of golf package for patient and concerns about responsibility towards patients

Other (please specify):_______________

**Section 5: There are different ways in which primary care patients could potentially be linked with the golf packages. These can include passive advertising, promotion of self-referral, formal referral or prescription, and can be direct from the GP practice to the people running the golf package or to a link worker/community connector.**

17. What, from your perspective, would be a good approach to linking eligible patients to a community-based golf package from the GP practice?

[text box]_____________________

18. What feedback, if any, would you like to receive feedback on a patient’s referral/connection to a community-based golf package?

[text box]_________________

19. How, if at all, would you like to receive feedback on a patient’s referral/connection to a community-based golf package?

[text box]_____________________

**END OF SURVEY NOTE REGARDING STEP 2: CO-DESIGN SESSION**

We thank you for your participation in this research project.

Please provide an email address for us to send you a £5 digital thank you voucher for your participation. This will not be used for any other purpose.

________________________________________________________________________________

If you would like to find out more information about taking part in the next step of this research and are happy for us to contact you using the email address you have provided then please tick this box consenting for us to contact you with further information	⃣

**COMMUNITY LINK WORKERS SURVEY**

The aim of this research project is to design connection pathways from GP practices to golf packages offered by local golf clubs in Fife for adults who would benefit from improving their physical activity levels. **We are keen to hear your views on these golf packages and how GP patients can be connected with them.**

The packages offered by local clubs aim to increase physical activity levels and improve physical and mental health and wellbeing for those adults new to golf and those who have not played in some time. Access to equipment, appropriate coaching sessions and golf activities facilitated by a qualified coach will be provided. A buddy scheme will be central to these packages to help participants feel supported. Social events will allow the participants to build bonds and feel part of the club. These buddy and social links will help to encourage participants to continue with their golf experience once the package has ended. Clubs may also offer participants access to follow-on sessions or activities and membership offers to maintain their engagement.

We would be grateful if you would answer the following questions. You are free to leave any question that you don’t wish to answer.

**Section 1: We want to make sure we get a wide range of perspectives. Therefore, we will ask you a few questions about yourself and your experiences.**

3. What is your professional role?

Local area co-ordinator

Community connector

Volunteer

Other: _______________

1. How long have you held that role?

Less than 1 year

1-4years

5-9years

10+ years

4. What is your age (years)?

18-24

25-34

35-44

45-54

55-64

65-74

75+

20. How would you describe your gender?

Female

Male

Other gender identity: ___________________________

Prefer not to answer

**Section 2: The packages offered by local clubs aim to increase physical activity levels and improve physical and mental health and wellbeing for those adults new to golf and those who have not played in some time. Access to equipment, appropriate coaching sessions and golf activities facilitated by a qualified coach will be provided. A buddy scheme will be central to these packages to help participants feel supported. Social events will allow the participants to build bonds and feel part of the club. These buddy and social links will help to encourage participants to continue with their golf experience once the package has ended. Clubs may also offer participants access to follow-on sessions or activities and membership offers to maintain their engagement.**

21. What do you think should be included in the golf package to achieve the aim of increasing physical activity levels and improving physical and mental health and wellbeing? [you can select multiple options]

group coaching session

putting activities

driving range activities

buddy scheme

social activities/events

provision of golf equipment during package

other:____________

22. What do you think should be provided after the golf package is finished? [you can select multiple options]

follow-on golf sessions

follow-on invites to social events

membership offers

other: ___________

**Section 3: There are several potential ways in which people could be linked with the golf packages. One challenge is identifying the people who would benefit most from this programme i.e. those who are physically inactive and would benefit from increasing their activity level**

23. How, if at all, do you assess an individual’s suitability to take part in any of the physical activity opportunities that you may pass them/link them to?

[text box]________________________

24. The Scottish Physical Activity Screening Questionnaire (Scot-PASQ) is a standardised tool that provides a framework for meaningful physical activity conversations between health or social care professionals and people in their care. It helps identify how active someone is and informs what physical activity support is needed [add link to doc].

Are you familiar with this tool?

I have never seen it before

I have seen it but not used it myself in my professional role

I have used it in my professional role to identify people’s activity levels

Other: [text box]_____________________

25. Do you think you could use this tool in your role to help identify if someone is suitable for taking part in a physical activity opportunity that you are linking/passing them on to?

Yes

No

Unsure

Please provide a brief reason for choice:[text box]________________________

26. In what form might you be able to use the Scot-PASQ screening tool?
    1. electronic form
    2. paper form
    3. as a conversation example guide (i.e. not followed word for word or fully completed)
    4. other: _______

Please give a brief reason for your choice. [text box]________________________

27. Do you think it would work to invite patients to fill out the Scot-PASQ themselves?

Yes

No

Unsure

Please provide a brief reason for choice:[text box]________________________

28. Do you have any other thoughts or ideas about how to identify physically inactive patients who might benefit from a community-based golf package?

[text box]________________________

**Section 4: There are different ways in which people could be encouraged and helped to increase their physical activity levels by taking part in a community-based golf package. We keen to hear your views about this.**

29. What, in your experience, are effective communication strategies for promoting physical activity with patients?

[text box]_____________________

30. Would you be happy to discuss a community-based golf package for physical activity with individuals referred/signposted to you?

Yes

No

Unsure

i. If unsure, please provide a brief reason:[text box]________________

31. Do you have any suggestions and ideas about what techniques could be helpful for trying to motivate a patient to change their physical activity behaviour and then getting them to turn motivation into action?

[text box]________________________

**Section 5: There are several potential ways in which GP patients could be linked with the golf packages. We would appreciate your views about this.**

32. In your current role, how are individuals currently passed on to you for linking to opportunities in the community? [please select all that apply]

GP/health professional completes form with patients and sent to us in post

GP/health professional completes form with patients and sends via email

GP/health professional completes form with patients online and sent to us electronically through the system

GP/health professional calls you with patient details

Patient gets letter of referral to our service and they contact us themselves

Patient gets letter of referral to our service and if they opt in/don't opt out then their details are passed to us

Other: ____________________

i. What works well/does not work well?

[text box]________________________

ii. What other approaches, if any, are you aware of that are used to link individuals/patients to community opportunities, e.g. in other health boards?

[text box]________________________

33. If patient details were passed on from GP practice to you as the co-ordinator/connector, what format would work best?
    i. electronic form
    ii. paper form
    iii. telephone call
    iv. other: _______

Please give a brief reason for your choice[text box]________

34. What format would work best for you for you to then connect patients to a community-based golf package?
    1. electronic form
    2. paper form
    3. telephone call
    4. other: _______

Please give a brief reason for your choice[text box]________

35. Are there any other ways in which you think the patient link-up to a community-based golf package could work?

[text box]________________________

36. What feedback, if any, would you like to receive feedback on a patient’s referral/connection to a community-based golf package?

[text box]_________________

37. How, if at all, would you like to receive feedback on a patient’s referral/connection to a community-based golf package?

[text box]________________________

**END OF SURVEY NOTE REGARDING STEP 2: CO-DESIGN SESSION**

We thank you for your participation in this research project.

Please provide an email address for us to send you a £5 digital thank you voucher for your participation. This will not be used for any other purpose.

________________________________________________________________________________

If you would like to find out more information about taking part in the next step of this research and are happy for us to contact you using the email address you have provided then please tick this box consenting for us to contact you with further information	⃣

**GOLF CLUB STAFF & VOLUNTEERS SURVEY**

The aim of this research project is to design connection pathways from GP practices to golf packages offered by local golf clubs in Fife for adults who would benefit from improving their physical activity levels. **We are keen to hear your views on these golf packages and how GP patients can be connected with them.**

The packages offered by local clubs aim to increase physical activity levels and improve physical and mental health and wellbeing for those adults new to golf and those who have not played in some time. Access to equipment, appropriate coaching sessions and golf activities facilitated by a qualified coach will be provided. A buddy scheme will be central to these packages to help participants feel supported. Social events will allow the participants to build bonds and feel part of the club. These buddy and social links will help to encourage participants to continue with their golf experience once the package has ended. Clubs may also offer participants access to follow-on sessions or activities and membership offers to maintain their engagement.

We would be grateful if you would answer the following questions. You are free to leave any question that you don’t wish to answer. **Please note**, you are not signing up your golf club to host a golf package. We are looking for your views.

**Section 1: We want to make sure we get a wide range of perspectives. Therefore, we will ask you a few questions about yourself and your experiences.**

5. What is your role at the golf club?

Golf club manager/assistant manager

Golf professional

Volunteer at golf club

Other: _______________

1. How long have you held that role?

Less than 1 year

1-4 years

5-9 years

10+ years

6. What is your age (years)?

18-24

25-34

35-44

45-54

55-64

65-74

75+

38. How would you describe your gender?

Female

Male

Other gender identity (please specify) ___________________________

Prefer not to answer

**Section 2: The community-based golf packages will be designed for any patient who is over 18 years who would benefit from improving their physical activity level and are new to golf or have not been active with golf for some time.** **There are different ways that people could be linked with one of the golf packages. One way is through their GP practice. A challenge of this is identifying the people who would benefit most from the golf packages.**

39. If you/your golf club were to be a part of hosting these packages, are there any health conditions that you think would not be suitable to be connected by health professionals/GP practices? Why?

[text box] _______________

**Section 3: The packages offered by local clubs aim to improve physical health and wellbeing for those adults new to golf and those who have not played in some time. Access to equipment, appropriate coaching sessions and golf activities facilitated by a qualified coach will be provided. A buddy scheme will be central to these packages to help participants feel supported. Social events will allow the participants to build bonds and feel part of the club. These buddy and social links will help to encourage participants to continue with their golf experience once the package has ended. Clubs may also offer participants access to follow-on sessions or activities and membership offers to maintain their engagement in golf.**

40. What do you think should be included in the golf package to achieve the aim of increasing physical activity levels and improving physical and mental health and wellbeing? [you can select multiple options]

group coaching session

putting activities

driving range activities

buddy scheme

social activities/events

provision of golf equipment during package

other:____________

41. What do you think should be provided after the golf package is finished? [you can select multiple options]

follow-on sessions once package is complete

follow-on invites to social events once package is complete

membership offers

other: ___________

42. What things do you think would be easy for a golf club to include in the community-based golf package for patients?

[text box] _______________

i. What would the clubs need to do this?

[text box] _______________

43. What things do you think would be difficult for a golf club to include in the community-based golf package for patients? Why?

[text box] _______________

44. How would being involved in offering a community-based golf package be of benefit to a club?

[text box] _______________

45. Would your club have capacity for such a package in terms of course use, practice facility, staff and volunteers? **Please note:** we are not asking for your club to sign up to host a community-based golf package

Yes

No

**Section 4:** **There are different ways in which people could potentially be linked with the golf package through the GP practice. Patients could find out about the golf package on offer through an advert in the practice, a letter from a health professional, completing a referral from etc.**

46. From your perspective, would it better if:

A. an eligible patient contacted the people running the golf package at the club, or

B. the people running the package at your club received the patient’s contact details and contacted them about the golf package?

Please select your preference of A or B and give a reason for choice [text box]________

47. If a patient’s contact details were passed on to the people running the golf package at your club, what format do you think would work best? [Please select one option]
    1. electronic form
    2. paper form
    3. telephone call
    4. other: _______

Please give a brief reason for your choice [text box]________

48. Do you have any other thoughts about community-based golf packages to increase physical activity levels?

[text box]________

**END OF SURVEY NOTE REGARDING STEP 2: CO-DESIGN SESSION**

We thank you for your participation in this research project.

Please provide an email address for us to send you a £5 digital thank you voucher for your participation. This will not be used for any other purpose.

If you would like to find out more information about taking part in the next step of this research and are happy for us to contact you using the email address you have provided then please tick this box consenting for us to contact you with further information	⃣
